# Polymorphisms within the *RANK* and *RANKL* Encoding Genes in Patients with Rheumatoid Arthritis: Association with Disease Progression and Effectiveness of the Biological Treatment

**DOI:** 10.1007/s00005-020-00590-6

**Published:** 2020-08-19

**Authors:** Joanna Wielińska, Katarzyna Kolossa, Jerzy Świerkot, Marta Dratwa, Milena Iwaszko, Bartosz Bugaj, Barbara Wysoczańska, Monika Chaszczewska-Markowska, Sławomir Jeka, Katarzyna Bogunia-Kubik

**Affiliations:** 1 Laboratory of Clinical Immunogenetics and Pharmacogenetics, Hirszfeld Institute of Immunology and Experimental Therapy, Polish Academy of Sciences, Wroclaw, Poland; 2 Department of Rheumatology and Connective Tissue Diseases, Jan Biziel University Hospital No. 2, Bydgoszcz, Poland; 3 Department of Rheumatology and Internal Medicine, Wroclaw Medical University, Wroclaw, Poland; 4 Ludwik Rydygier Collegium Medicum in Bydgoszcz, Nicolaus Copernicus University, Torun, Poland

**Keywords:** RANK, RANKL, Polymorphism, Anti-TNF therapy, Rheumatoid arthritis

## Abstract

Inconsistency of the results regarding the genetic variability within genes coding for receptor activator of nuclear factor κB (RANK) and its ligand (RANKL) in rheumatoid arthritis (RA) prompted us to study the *RANK* and *RANKL* polymorphisms as potential biomarkers associated with disease predisposition and response to anti-TNF treatment in a group of Polish patients with RA. This study enrolled 318 RA patients and 163 controls. *RANK* (rs8086340, C > G; rs1805034, C > T) and *RANKL* (rs7325635, G > A; rs7988338 G > A) alleles were determined by real-time PCR with melting curve analysis and related with clinical parameters. In addition, RANKL serum levels were measured by ELISA. The *RANK* rs8086340-*G* allele was overrepresented among patients as compared to controls (OD = 1.777, *p* = 0.038). C-reactive protein (CRP) levels were significantly (*p* < 0.05) associated with *RANK* rs8086340 polymorphism and were higher in the *CC*-homozygotes at the baseline while lower in the *GG*-carriers at the 12th week of the treatment. At the latter time point *RANKL* rs7325635-*GG-*positive patients also showed significantly lower CRP concentrations. Higher alkaline phosphatase levels before induction of anti-TNF therapy were observed in *RANK* rs8086340 and *RANK* rs1805034 *CC* homozygotes (*p* = 0.057 and *p* = 0.035, respectively). The *GG* homozygosity of both *RANKL* single nucleotide polymorphisms was significantly associated with the number of swollen joints (rs7988338 and rs7325635, before and at the 12th week of therapy, respectively, *p* < 0.05 in both cases). These results imply that polymorphisms within the *RANK* and *RANKL* genes affect RA susceptibility and anti-TNF treatment outcome.

## Introduction

Rheumatoid arthritis (RA) is an autoimmune disorder that is present in approximately 1% of the Caucasian population, mostly women. It is identified by inflammation of joint synovial membrane, leading to the progression of cartilage and bone tissue damage, eventually prompting disability. RA is caused by a combination of environmental, genetic and stochastic factors (Smolen et al. 2016). Introduction of the tumor necrosis factor (TNF) inhibitors to RA treatment had a positive effect on the quality of patients’ lives. Biologic agents successfully suppress disease symptoms, as well as stop further bone damage and reduce disability (Geiler et al. 2011). Our previous studies showed that some genetic features may influence disease susceptibility or anti-TNF therapy outcome in RA patients (Bogunia-Kubik et al. 2015; Gębura et al. 2017; Iwaszko et al. 2018; Świerkot et al. 2015a).

Well-characterized risk factors and early diagnosis are crucial for preventing bone loss and achieving therapeutic success. However, molecular mechanisms behind osteoporosis in RA have not been fully elucidated. It is known that glucocorticoid therapy, inflammatory cytokines, reduced physical activity and low calcium intake are involved in RA-related osteoporosis (Corrado et al. 2017). Pathak et al. (2014) demonstrated that sera from patients with active disease enhanced osteoblast proliferation and differentiation via receptor activator of nuclear factor (NF)-κB ligand (RANKL), thus leading to bone loss. Dysregulation of balance between bone resorption and formation was previously observed in RA (Boyce and Xing 2008). Under chronic inflammation conditions, and in presence of proinflammatory cytokines such as interleukin (IL)-1, TNF-α, IL-6 and IL-17, RANKL is expressed by T cells, fibroblast-like synoviocytes, bone marrow stromal cells (Braun and Zwerina 2011; Schett 2011) and B cells, thereby promoting osteoclast-dependent bone resorption (Meednu et al. 2016; Yeo et al. 2011). RANKL plays a pivotal role in osteoclast activation, development and survival (Nemeth et al. 2011). Nevertheless, binding to its receptor (RANK) is required for the process to begin. RANK is a transmembrane protein belonging to the TNF receptor superfamily (TNFRSF) (Schett et al. 2005) and is mainly expressed on the surface of the macrophage/monocyte lineage cells. Besides that, it is widely present on the surface of osteoclast progenitors and mature osteoclasts (Liu and Zhang 2015). Typically those molecules do not have kinase activity and must engage factors able to activate signaling pathways (Walsh and Choi 2014). During osteoclastogenesis, the TNF receptor-associated factors are recruited, and NF-κB, an essential transcription factor, is activated (Leibbrandt and Penninger 2008). The nature of the relationship between osteoclasts and TNF-family proteins has been reported in numerous studies (Kitaura et al. 2013; Ono and Nakashima 2018).

In our study, we focused on the genetic variability of the *RANK* (*TNFRSF11A*) and *RANKL* (*TNFSF11*) genes in RA patients subjected to anti-TNF treatment.

The significance of chosen *RANK* and *RANKL* single nucleotide polymorphisms (SNPs) have been described before. The role of *RANK* rs1805034 polymorphism has been analyzed in regard to RA (Mohamed et al. 2016; Yang et al. 2019), cancer (Yin et al. 2014; Zhang et al. 2015), hip osteoporotic fractures (Zhang et al. 2011), knee osteoarthritis (Wang et al. 2019), age at menarche (Duan et al. 2015; Pan et al. 2012) and in Paget’s disease (Chung et al. 2010). The *RANK* rs8086340 was also previously documented in RA patients, as well as with respect to age at natural menopause (Lu et al. 2010). It has been reported that *RANKL* rs7325635 may be a genetic marker in heart failure patients (Schmitz et al. 2014) and *RANKL* rs7988338 was significantly associated with femoral neck compression strength index (Dong et al. 2009). However, according to our knowledge, those polymorphisms has never been studied as potential markers associated with disease predisposition and response to treatment with TNF inhibitors in an independent cohort of Polish RA patients.

## Materials and Methods

### Patients and Controls

Three-hundred-eighteen Polish patients with diagnosed rheumatoid arthritis and qualified for treatment with anti-TNF agents were enrolled in this study. Patients were classified according to the 2010 American College of Rheumatology criteria, as well as by the presence of active disease represented by the Disease Activity Score in 28 joints ([DAS28] ≥ 5.1) prior to initiating biologic agent therapy. The following inclusion criteria were applied: age over 18 years, a complete medical history and physical examination of patients and resistance to treatment with at least two disease-modifying anti-rheumatic drugs. The exclusion criteria included clinically significant impairment of hepatic and renal function, the coexistence of other systemic diseases of connective tissue besides RA, infection with hepatotropic viruses; infections resistant to therapy; ongoing history of cancer or uncontrolled diabetes, alcohol abuse, pregnancy or breastfeeding, insufficient clinical records, and unwillingness or inability to cooperate. Data collected from the patients comprise: level of C-reactive protein (CRP), presence of rheumatoid factor (RF), presence of anti-cyclic citrullinated peptide (anti-CCP) antibodies, disease activity score (DAS28), and erythrocyte sedimentation rate (ESR), painful and swollen joint counts, global health assessments by a patient and a physician, and visual analogue scale (VAS) of pain value. The study was approved by the Wrocław Medical University Ethics Committee (Identification Code KB-625/2016) and written informed consent was obtained from all participants. Baseline characteristics of the patients are summarized in Table 1. The control group consisted of 163 Polish unrelated healthy blood donors with no personal history of autoimmune diseases.

**Table 1:** Characteristics of RA patients

Number of RA patients	318
Females/males (% of females)	248/70 (78.0%)
Age (years) [mean ± SD]	51.3 (± 12.9)
Disease onset (years) [mean ± SD]	39.3 (± 12.2)
Disease duration (years) [mean ± SD]	12.4 (± 8.27)
DAS28 baseline [mean ± SD]	6.52 (± 0.64)
CRP baseline [mean ± SD]	23.6 (± 35.0)
RF-positive [%]	66.8%
Anti-CCP positive [%]	93.8%

*DAS28* disease activity score, *CRP* C-reactive protein, *RF* rheumatoid factor, *anti-CCP* anti-cyclic citrullinated peptide antibodies

A subgroup of 107 patients was characterized by the following additional parameters: levels of calcium, alkaline phosphatase (ALP), vitamin D3 and thyroid-stimulating-hormone (TSH) that together with the number of painful and swollen joints, and VAS score might correlate with bone condition.

### Anti-TNF Therapy Outcome

Disease activity in RA patients was estimated using the DAS28 score based on four components: number of swollen and tender joints, levels of CRP and ESR, patients’ global assessment of general health expressed on the visual analogue scale. The level of disease activity was interpreted as either low (DAS28 ≤ 3.2), moderate (3.2 < DAS28 ≤ 5.1), or high (DAS28 > 5.1). Clinical response was assessed according to the European League Against Rheumatism (EULAR) criteria at the 12th week after initiation of anti-TNF treatment. The patients were divided into three groups according to their response to treatment: good, moderate, or non-responders. A good response was defined as improvement in the DAS28 score, specifically ΔDAS28 > 1.2 and DAS28 at endpoint ≤ 3.2 at the endpoint. The moderate response was defined as either ΔDAS28 > 1.2 and DAS28 at endpoint > 3.2 or 0.6 < ΔDAS28 ≤ 1.2 and DAS28 at endpoint ≤ 5.1. A lack of response was defined as ΔDAS28 ≤ 0.6 or 0.6 < ΔDAS28 ≤ 1.2 and DAS28 at endpoint > 5.1, as previously described (Iwaszko et al. 2018).

### SNP Selection and Genotyping

For the present study, the genetic variants of the *TNFRSF11A* and *TNFSF11* genes were selected based on available literature analysis as well as search results from NCBI Database of Short Genetic Variations (dbSNP). Information regarding predicted functional consequences of SNPs was obtained using the SNPinfo Web Server (Xu and Taylor 2009). The *RANK* gene is located on chromosome 18. rs8086340 (C > G) is placed within intron 1 and rs1805034 (C > T) missense substitution in exon 6 leads to an amino acid change from alanine to valine. The *RANKL* gene is located on chromosome 13 and both rs7325635 (G > A) and rs7988338 (G > A) are situated in intron 2 in possible transcription factor binding sites. The minor allele frequencies (MAF) of all the selected SNPs were higher than 0.15.

Genomic DNA was isolated from peripheral blood of RA patients and controls using QIAamp DNA Blood Midi Kit (Qiagen, Hilden, Germany) according to recommendations of the manufacturer. *RANK* (rs8086340, rs1805034) and *RANKL* (rs7325635, rs7988338) alleles were determined by real-time polymerase chain reaction (PCR) amplification and melting-curve analysis using LightSNiP assay (TIB MOLBIOL, Berlin, Germany) on the LightCycler 480 Real-Time PCR system (Roche Diagnostics, Rotkreuz, Switzerland).

### RANKL Serum Level Analysis

Serum concentration of RANKL was measured by commercial ELISA kits (DY626, R&D Systems, Minneapolis, MN, USA) according to protocols provided by the manufacturer. The analyses were performed for a subgroup of RA patients consisting of 109 individuals before anti-TNF treatment and 99 controls. The absorbance was measured in a Tecan Sunrise absorbance reader and Magellan software (Tecan Trading AG, Switzerland). The optical density of each well run in duplicate was determined by microplate reader set to 450 nm with wavelength correction set to 570 nm. Peptide concentration in the samples was measured by comparing the optical density of the sample with a computer-generated four parameters logistic curve-fit standard curve.

### Statistical Analysis

The Hardy–Weinberg equilibrium was tested in patients and controls for each SNP. Potential associations between examined SNPs and clinical parameters of RA patients were analyzed applying the Mann–Whitney *U* test for quantitative data and the Fisher’s exact test for parametric values. The frequencies of respective genotype groups among RA patients in relation to the anti-TNF outcome were investigated by comparing the EULAR scores using Fisher’s exact test. Fisher’s exact test was also used to compare genotype variation distribution within patients and controls. *p* values less than 0.05 were considered statistically significant. All statistical calculations were performed in the GraphPad7 Prism software.

## Results

### Distribution of RANK and RANKL Genotypes in Patients and Controls

Genotype distribution of all the studied SNPs in both groups (patients and controls) are presented in Table 2. MAF values were as follows: *RANK* rs8086340 C: 0.361 (patients) vs. 0.383 (controls), *RANK* rs1805034 *C*: 0.445 vs. 0.470, *RANKL* rs7325635 *A*: 0.461 vs. 0.460, *RANKL* s7988338 *A*: 0.168 vs. 0.190.

**Table 2:** Distribution of *RANK* (rs8086340, C > G; rs1805034, C > T) and *RANKL* (rs7325635, G > A; rs7988338 G > A) genotypes in RA patients and controls

Gene	Genotype	RA patients *N* (%)	Controls *N* (%)
*RANK* rs8086340	*CC*	37 (11.7%)^a^	31 (19.0%)
	*CG*	155 (48.9%)^b^	63 (38.7%)
	*GG*	125 (39.4%)	69 (42.3%)
*RANK* rs1805034	*CC*	65 (20.4%)	37 (22.7%)
	*CT*	153 (48.1%)	80 (49.1%)
	*TT*	100 (31.5%)	46 (28.2%)
*RANKL* rs7325635	*GG*	98 (30.8%)	44 (27.2%)
	*GA*	147 (46.2%)	87 (53.7%)
	*AA*	73 (23.0%)	31 (19.1%)
*RANKL* *r*s7988338	*GG*	219 (68.9%)	107 (65.6%)
	*GA*	91 (28.6%)	50 (30.7%)
	*AA*	8 (2.52%)	6 (3.63%)

*N* number of individuals with a given genotype

^a^OR = 1.777, 95% CI (1.069–3.010), *p* = 0.038

^b^OR = 1.519, 95% CI (1.039–2.227), *p* = 0.034

Genotype frequencies of the *RANK* rs8086340 polymorphism in RA patients were different from those in the control group (Table 2). Both the presence of the *G* variant as well as *CG* heterozygosity were more frequent among patients than controls. *RANK* heterozygous genotype was present in 48.9% of patients and 38.7% of controls (OR = 1.519, 95% CI (1.039–2.227), *p* = 0.034). The *RANK* rs8086340 *G* allele was detected in 88.3% (280/317) of RA patients, as compared to 81.0% (132/163) of controls (*CG* + *GG* vs. *CC*, OR = 1.777, 95% CI (1.069–3.010); *p* = 0.038). These results imply that the *RANK* rs8086340 SNP may affect disease susceptibility.

There were no differences in genotype distribution of *RANK* rs1805034, *RANKL* rs7325635, *RANKL* rs7988338 between patients and controls.

### Relationship between Patients’ Genotypes and Clinical Parameters

Among the 318 patients included in this study, the mean (± SD) of the CRP concentration in RA patients’ blood was 23.6 (± 35.0) and the disease activity score (DAS28) level was 6.52 (± 0.64). After 12 weeks of anti-TNF treatment, both parameters significantly decreased, to 9.84 (± 16.6) and 4.45 (± 1.05), respectively, (*p* < 0.0001 in both cases).

Except for CRP levels, none of the clinical parameters such as RF, anti-CCP or DAS28 was found to be associated with any of the polymorphisms studied. For details, please see Table 3. Significant differences were observed between levels of CRP and *RANK* rs8086340 and *RANKL* rs7325635 SNPs. In general (both before, and after treatment with TNF inhibitors), CRP concentrations were lower in the *RANK* rs8086340 *GG* and *RANKL* rs7325635 *GG* homozygotes, as compared to patients carrying other *RANK* rs8086340 or *RANKL* rs7325635 genotypes, respectively. These differences were especially noticeable after the 12th week of anti-TNF administration (*RANK* rs8086340: GG vs. CG + CC, *p* = 0.025; *RANKL* rs7325635: GG vs. GA + AA; *p* = 0.022).

**Table 3:** Distribution of the *RANK* and *RANKL* genotypes with respect to selected clinical parameters (at baseline and at the 12th week of the therapy)

Gene		DAS28 at Baseline	DAS28 at 12th week	CRP at Baseline	CRP at 12th week	RF+	CCP+
		Mean (± SD)	Mean (± SD)	Mean (± SD)	Mean (± SD)	Number (%)	Number (%)
*RANK rs8086340*	*CC*	6.56 (± 0.76)	4.44 (± 1.11)	31.1^a^ (± 34.4)	12.7 (± 20.6)	19 (61.3%)	27 (93.1%)
	*CG*	6.49 (± 0.64)	4.48 (± 1.01)	24.9 (± 38.2)	11.3 (± 18.5)	94 (66.7%)	124 (95.4%)
	*GG*	6.54 (± 0.60)	4.45 (± 1.05)	20.0 (± 30.4)	7.15^b^ (± 11.5)	77 (68.1%)	91 (91.9%)
*RANK rs1805034*	*CC*	6.59 (± 0.61)	4.55 (± 0.95)	22.42 (± 34.65)	8.21 (± 12.99)	41 (71.9%)	44 (91.7%)
	*CT*	6.46 (± 0.68)	4.37 (± 1.10)	23.86 (± 38.9)	9.76 (± 17.2)	93 (67.9%)	122 (93.1%)
	*TT*	6.57 (± 0.59)	4.52 (± 0.99)	24.1 (± 28.7)	11.0 (± 17.7)	57 (61.9%)	77 (96.3%)
*RANKL rs7325635*	*GG*	6.54 (± 0.60)	4.39 (± 1.11)	19.8 (± 30.7)	7.17^c^ (± 11.7)	57 (65.5%)	73 (96.1%)
	*GA*	6.52 (± 0.68)	4.42 (± 0.94)	24.3 (± 32.4)	10.8 (± 16.6)	84 (64.6%)	109 (91.6%)
	*AA*	6.49 (± 0.62)	4.59 (± 1.12)	27.3 (± 43.9)	11.4 (± 21.0)	50 (72.5%)	61 (95.3%)
*RANKL rs7988338*	*GG*	6.52 (± 0.65)	4.52 (± 0.99)	24.5 (± 38.5)	9.60 (± 16.3)	136 (68.3%)	166 (92.7%)
	*GA*	6.55 (± 0.63)	4.28 (± 1.16)	22.7 (± 25.7)	10.6 (± 17.7)	52 (64.2%)	71 (95.9%)
	*AA*	6.33 (± 0.52)	4.62 (± 0.71)	7.50 (± 6.58)	8.38 (± 8.22)	3 (50.0%)	6 (100%)

^a^*p* = 0.036

^b^*p* = 0.025

^c^*p* = 0.022

Additionally, *RANK* rs8086340 *CC* carriers had significantly higher CRP levels before treatment than carriers of other genotypes of this SNP (CG + GG vs. CC; *p* = 0.036).

### Response to Anti-TNF Therapy

Response to anti-TNF therapy after 12 weeks was evaluated using EULAR criteria. In general, a good and moderate response was achieved by 12.2% and 80.0% of patients, respectively, while the remaining 7.8% of RA patients investigated did not respond to treatment. The studied polymorphisms were not found to affect the outcome of biological treatment.

### Relationship between Patients’ Genotypes and Bone Parameters or Thyroid Dysfunction

We were able to analyze and compare the distribution of selected SNPs analyzed in the present study with regard to various bone parameters such as calcium and alkaline phosphatase levels, vitamin D, TSH levels (Table 4), number of painful and swollen joints and VAS score (Table 5).

**Table 4:** Distribution of the *RANK* and *RANKL* genotypes with regard to biochemical parameters level presented as mean (± SD)

Gene	Genotype	Calcium [mg/dl]	Alkaline phosphatase [U/l]	Vitamin D3 [ng/ml]	TSH [ulU/ml]
*RANK rs8086340*	*CC*	9.59 (± 0.42)	104.7 (± 65.9)^a^	23.9 (± 9.65)	1.52 (± 1.38)
	*CG*	9.57 (± 0.42)	72.3 (± 21.0)	20.3 (± 8.07)	1.27 (± 0.72)
	*GG*	9.58 (± 0.40)	81.4 (± 32.5)	22.7 (± 11.1)	1.36 (± 0.92)
*RANK rs1805034*	*CC*	9.56 (± 0.26)	92.3 (± 32.5)^b^	20.2 (± 6.15)	1.32 (± 0.72)
	*CT*	9.67 (± 0.39)	78.2 (± 42.3)	20.1 (± 8.46)	1.43 (± 0.73)
	*TT*	9.51 (± 0.48)	77.3 (± 29.7)	24.8 (± 12.3)	1.28 (± 1.15)^c^
*RANKL rs7325635*	*GG*	9.57 (± 0.40)	80.9 (± 42.3)	20.2 (± 9.39)	1.31 (± 0.93)
	*GA*	9.51 (± 0.38)	76.8 (± 27.0)	21.4 (± 8.49)	1.40 (± 0.69)
	*AA*	9.76 (± 0.43)	89.7 (± 38.4)	27.4 (± 13.4)	1.32 (± 1.25)
*RANKL rs7988338*	*GG*	9.61 (± 0.43)	80.2 (± 29.7)	22.4 (± 10.2)	1.39 (± 0.99)
	*GA*	9.49 (± 0.35)	83.0 (± 52.0)	22.0 (± 9.93)	1.19 (± 0.69)
	*AA*	9.80 (± 0.26)	82.0 (± 13.1)	15.5 (± 0.00)	1.55 (± 0.64)

^a^*p* = 0.057

^b^*p* = 0.035

^c^*p* = 0.052

**Table 5:** Distribution of the *RANK* and *RANKL* genotypes with respect to the number of painful or swollen joints and visual analogue scale (VAS) for pain score presented as mean (± SD) (at baseline and at the 12th week of the therapy)

Gene		Number of painful joints at baseline	Number of painful joints at 12th week	Number of swollen joints at baseline	Number of swollen joints at 12th week	VAS [mm] at baseline	VAS [mm] at 12th week
*RANK rs8086340*	*CC*	15.9 (± 6.30)	9.64 (± 5.61)	11.4 (± 6.26)	6.00 (± 5.49)	76.7 (± 11.6)	44.9 (± 17.4)
	*CG*	16.3 (± 5.14)	9.29 (± 6.38)	12.6 (± 5.82)	7.00 (± 6.94)	76.7 (± 12.3)	48.9 (± 17.0)
	*GG*	18.2 (± 5.60)	8.87 (± 5.46)	12.8 (± 5.80)	5.50 (± 4.71)	75.2 (± 9.49)	46.9 (± 19.4)
*RANK rs1805034*	*CC*	18.1 (± 5.69)	8.86 (± 5.72)	12.4 (± 5.93)	5.24 (± 4.57)	75.4 (± 12.1)	49.2 (± 14.7)
	*CT*	16.3 (± 5.06)	8.71 (± 6.25)	11.8 (± 5.74)	6.40 (± 6.17)	74.4 (± 10.7)	44.6 (± 18.6)
	*TT*	17.6 (± 5.97)	9.83 (± 5.46)	13.4 (± 5.87)	6.57 (± 6.18)	78.1 (± 10.1)	49.6 (± 19.5)
*RANKL rs7325635*	*GG*	17.9 (± 5.09)	10.1 (± 5.79)	13.1 (± 6.34)	7.82 (± 6.41)^a^	76.1 (± 9.47)	47.2 (± 18.9)
	*GA*	17.1 (± 6.10)	8.51 (± 6.00)	12.4 (± 5.10)	4.49 (± 4.75)	75.7 (± 13.2)	44.3 (± 18.5)
	*AA*	16.0 (± 5.34)	8.41 (± 5.49)	11.6 (± 6.18)	6.35 (± 5.74)	76.2 (± 8.67)	54.3 (± 14.1)
*RANKL rs7988338*	*GG*	16.7 (± 5.60)	9.03 (± 5.60)	11.7 (± 5.59)^b^	5.90 (± 5.05)	75.0 (± 11.5)	49.4 (± 17.1)
	*GA*	18.4 (± 5.44)	9.17 (± 6.62)	14.4 (± 6.03)	6.48 (± 7.51)	79.0 (± 8.19)	43.2 (± 19.2)
	*AA*	20.0 (± 2.83)	12.0 (± 2.83)	17.5 (± 6.36)	11.0 (± 4.24)	74.0 (± 8.48)	35.5 (3 ± 4.6)

^a^*p* = 0.035

^b^*p* = 0.045

Analyses revealed that both polymorphisms in the *RANK* gene (rs8086340 and rs1805034) are associated with alkaline phosphatase level (Table 4), whereas *RANKL* variants correlated with the number of swollen joints before and after therapy (Table 5). Please note that both numbers of painful (17.20 ± 5.54) and swollen joints (12.52 ± 5.81), as well as VAS scores (75.98 ± 10.81) significantly decreased following the biological treatment, to 9.14 ± 5.81 and 6.17 ± 5.76, as well as 47.44 ± 18.05, respectively, (*p* < 0.001 in all cases).

The *RANK* (rs8086340 and rs1805034) *CC* homozygotes were characterized with higher alkaline phosphatase levels. The statistically significant relationship was observed for rs1805034 (*CC* vs. *CT* + *TT*; *p* = 0.035), while a strong tendency was seen in the *RANK* rs8086340 SNP (*CC* vs. *CG* + *GG*; *p* = 0.057).

The *RANKL* rs7988338 *GG* homozygotes were identified with a smaller number of swollen joints compared to patients carrying the *A* allele (*GG* vs. *AG* + *AA*; *p* = 0.045). Also, a significant correlation between a number of swollen joints and *RANKL* rs7325635 was observed at the 12th week of the anti-TNF therapy. Interestingly, *RANKL* rs7325635 *GG* homozygotes in this polymorphism had also more swollen joints after treatment (*GG* vs. *AG* + *AA*; *p* = 0.035).

In addition, *RANK* rs8086340 *C* allele was more commonly observed in a group of patients with higher TSH levels (*TT* vs. *CT* + *CC*; *p* = 0.052), although more *TT* homozygous patients had thyroid dysfunction (TSH level below or above the norm (0.4–4 ulU/ml)) (7/36 = 19.4%) vs. 2/55 = 3.64%; *p* = 0.026).

### RANKL Serum Concentration

Serum concentrations of RANKL were assessed in 109 RA patients before anti-TNF treatment, 38 patients after 12 weeks of treatment and 99 controls. It appeared that only 32 patients at baseline, 15 after treatment and 41 controls presented with RANKL concentrations exceeding the minimum standard curve point (78.1 pg/ml). No statistically significant difference was observed between RANKL serum levels of patients and controls. However, it was noticed that average protein concentration equaled 811.75 pg/ml and 682.34 pg/ml, for RA patients before induction of anti-TNF agents and controls, respectively. Furthermore, no correlation was found between *RANKL* genotypes and serum levels, either in patients or in controls.

Interestingly, after 12 weeks of anti-TNF treatment, RANKL serum levels decreased to an average serum concentration of 647.35 pg/ml similar to that observed for the control group (682.34 pg/ml).

## Discussion

In recent years, several studies investigated the association between the *RANK* (*TNFRSF11A*) and *RANKL* (*TNFSF11*) gene polymorphisms and the risk for RA development in different populations. However, the results coming from these studies were conflicting. For example, Mohamed et al. (2016) described *RANK* rs1805034 and *RANKL* rs9525641 non-synonymous polymorphisms as potential genetic risk factors for osteoporosis in postmenopausal women with RA. In other studies, *RANKL* rs9525641 was found to be associated with younger age at onset of RA disease (Tan et al. 2010; Wu et al. 2004). In German patients, the major allele of *RANKL* rs2277438, and a minor variant of *RANK* rs35211496 (both located within intronic regions) were described to increase the risk for RA (Assmann et al. 2010). Moreover, the *RANKL* rs2277438 *G* allele was found to be associated with radiographic progression of joint damage (Furuya et al. 2007). A very recent study of Yang et al. (2019) documented that *RANKL* rs2277438 polymorphism increased RA risk, and that the *RANK* rs1805034 SNP was not related to RA risk. On the other hand, Wang et al. (2019) found the *RANK* rs1805034 SNP to be associated with susceptibility to knee osteoarthritis.

Our present study was conducted to evaluate the role of the *RANK* (rs8086340, C > G; rs1805034, C > T) and *RANKL* (rs7325635, G > A; rs7988338 G > A) SNPs as potential diagnostic biomarkers associated with the RA risk in the Polish population, and prognostic biomarkers affecting the outcome of the biological treatment. To the best of our knowledge, the present study is the first one investigating relationships of these selected SNPs with clinical parameters in patients with RA.

Similarly to the results of Yang et al. (2019), our study showed that the *RANK* rs1805034 SNP was not found to be related with RA risk. However, we observed a higher frequency of *RANK* rs8086340 heterozygotes and *G* allele carriers among patients than in controls, showing that patients possessing the *RANK* rs8086340 *G* allele were more prone to RA development. Such an association between *RANK* rs8086340 SNP and RA susceptibility was not previously described.

Recently published results in French cohorts found the *RANK* rs8086340 SNP to be correlated with the presence of anti-CCP (Ruyssen-Witrand et al. 2016), but we did not observe such a correlation in our patients of Polish origin, either with anti-CCP or RF. Concerning some other clinical parameters, we did notice a statistically significant decrease in the level of CRP and DAS28 during therapy, but only changes in CRP serum concentrations were related to investigated polymorphisms. We observed that *GG* homozygosity of both *RANK* rs8086340 and *RANKL* rs7325635 was associated with lower CRP levels, especially after the 12th week of anti-TNF treatment. Our observations correspond with earlier studies considering the role of TNF blocker as an effective disease activity reducer (Kurz et al. 2015).

SNPs investigated in this study may have a regulatory role in gene transcription. Polymorphism rs8086340 of the *RANK* gene is located within an intronic region while rs1805034 results in an amino acid change from alanine to valine. *RANKL* rs7325635, as well as rs7988338, are situated in an intronic area in a transcription factor binding site. Therefore, we also measured serum RANKL concentration in patients at baseline, at the 12th week after induction of anti-TNF agents and in the control group.

Previous studies reported significantly increased RANKL levels in RA patients compared to controls and in anti-CCP-positive individuals (Boman et al. 2017). Higher RANKL concentration was also detected before the onset of RA (Johansson et al. 2017). Interestingly, in our study, we observed some differences in serum concentrations between patients before initiation of biological treatment and controls, with a higher average protein concentration in RA patients (811.75 pg/ml vs. 682.34 pg/ml in controls). However, this difference did not reach statistical significance.

No significant relationship was detected between RANKL concentration and the both SNPs within the RANKL encoding gene. Although an average RANKL concentration was over 300 pg/ml higher at the baseline in patients carrying the *RANK* rs8086340 *G* allele, found in the present study to be associated with an increased risk for RA development. Patients carrying the *G* allele presented with an average of 889.04 pg/ml RANKL in serum vs. 579.84 pg/ml for patients lacking this genetic variant.

As for *RANKL* SNPs investigated in our study, Ruyssen-Witrand et al. (2016) described *RANKL* rs7325635 as significantly associated with anti-CCP antibody presence and erosions, whereas *RANKL* rs7988338 was significantly associated with femoral neck compression strength index, a phenotypic parameter integrating bone density, bone size, and body size, having significant potential to improve hip fracture risk assessment (Dong et al. 2009).

In our group of RA patients, the *GG* homozygosity of both *RANKL* SNPs (rs7988338 and rs7325635) was significantly associated with the number of swollen joints while the *RANK* rs8086340 and rs1805034 *CC* homozygotes were significantly more frequent among patients with higher ALP levels. These relationships have not been previously described. Surprisingly, a significant difference was also noted with respect to glucocorticosteroid dose. The *RANKL* rs7988338 *G* allele carriers received a significantly lower dose of this drug than the *AA* homozygous patients (individual data not shown). Despite anti-inflammatory effects, glucocorticosteroids also affect the delayed progression of erosive lesions in the joints (Świerkot and Madej 2017). This finding is in line with our observation on the lower number of swollen joints before treatment in the *GG* homozygous patients.

We also observed a decrease in RANKL serum level during the therapy. At the 12th week of the treatment, the average protein concentration equaled 647.35 pg/ml which corresponds to RANKL level of the control group. This observation may reflect effectiveness of the anti-TNF treatment, which is in line with the results of González-Alvaro et al. (2007). They also showed a decrease in RANKL serum concentration during anti-TNF therapy, and further suggested that serum level of RANKL may be helpful in predicting outcome in patients receiving biologic agents (González-Alvaro et al. 2007). Also, Świerkot et al. (2015b) demonstrated decreasing RANKL concentration in the subgroup of RA patients with good/moderate MTX therapy response after 6 months of treatment.

The results of our study indicate a possible role of the *RANK* (*TNFRSF11A*) and *RANKL* (*TNFSF11*) gene polymorphisms as diagnostic and/or prognostic factors in Polish patients with RA. They imply that *RANK* rs8086340 SNP may affect disease susceptibility. Furthermore, *RANK* rs8086340 and *RANKL* rs7325635 polymorphisms may have a prognostic role in modulating the clinical outcome of anti-TNF therapy.

Nevertheless, further genetic studies on larger groups of patients of various origins subjected to biological treatment are necessary to confirm these findings.
